# Perioperative Dynamic Changes and Independent Associations of Pressure Pain Threshold in Trauma Patients: A Prospective Longitudinal Study

**DOI:** 10.1155/prm/8214001

**Published:** 2026-09-29

**Authors:** Zhijie Yan, Hao Li, Ziyu Wang, Wuzhen Su, Yibin Gao, Ruiyun Li, Chunwang Zhang, Zhicheng Gao, Hewei Wang, Jie Liu

**Affiliations:** ^1^ Department of Rehabilitation, The Third Affiliated Hospital of Henan Medical University, No. 599 Hualan Avenue Hongqi District, Xinxiang, Henan, China; ^2^ School of Rehabilitation, Henan Medical University, No. 601 Jinsui Avenue Hongqi District, Xinxiang 453000, Henan, China; ^3^ Department of Orthopaedics, Trauma Center, The Third Affiliated Hospital of Henan Medical University, No. 599 Hualan Avenue Hongqi District, Xinxiang 453000, Henan, China, traumacenter.org; ^4^ Department of Anesthesiology and Perioperative Medicine, The Third Affiliated Hospital of Henan Medical University, No. 599 Hualan Avenue Hongqi District, Xinxiang 453000, Henan, China; ^5^ Department of Rehabilitation, Huashan Hospital, Fudan University, No. 12 Middle Urumqi Road Jing’an District, Shanghai 200040, China, fudan.edu.cn; ^6^ Henan Medical Key Laboratory for Research of Trauma and Orthopaedics, The Third Affiliated Hospital of Henan Medical University, No. 599 Hualan Avenue Hongqi District, Xinxiang Henan, 453000, China; ^7^ Clinical Medical Centre of Tissue Engineering and Regeneration, Henan Medical University, No. 599 Hualan Avenue Hongqi District, Xinxiang 453000, Henan, China; ^8^ UBC Emeritus College, The University of British Columbia, Brock Commons South, 6180 Walter Gage Road, Vancouver V6T 1K2 British Columbia, Canada, ubc.ca

**Keywords:** linear mixed-effects models, orthopaedic trauma, pain sensitivity, perioperative pain, pressure pain threshold

## Abstract

**Background:**

Pressure pain threshold (PPT) is an objective quantitative sensory measure and intermediate biomarker of pain sensitivity. However, the dynamic perioperative alterations and independent clinical determinants of PPT remain insufficiently characterized.

**Materials and Methods:**

This prospective observational study included 67 adult patients undergoing orthopaedic trauma surgery. Bilateral PPT of injured and uninjured sides was measured at preoperative baseline and postoperative days (POD) 1–3 using a calibrated electronic algometer. Linear mixed‐effects models were applied to analyze PPT temporal trends, bilateral differences, and independent associations, with a significance level set at 0.05.

**Results:**

Continuous bilateral PPT reduction was detected throughout the early postoperative phase. The injured side exhibited significantly lower PPT than the uninjured side on POD 1 (mean difference = −2.43 N, 95% CI: −4.08 to −0.77, *p* = 0.004), whereas no significant bilateral differences were found preoperatively (*p* = 0.154), on POD 2 (*p* = 0.583), or on POD 3 (*p* = 0.523). No significant time‐by‐side interaction effects were identified during follow‐up. Multivariate analysis demonstrated that age (*β* = 0.116, *p* = 0.019) positively correlated with PPT, while female sex (*β* = −4.715, *p* = 0.003) and elevated body mass index (BMI) (*β* = −0.441, *p* = 0.022) independently decreased PPT values. In contrast, no statistically significant association with PPT was observed for injury type, surgical approach, and anesthesia regimen in the adjusted model. Sensitivity analyses adjusting for morphine equivalent dose, numeric rating scale pain scores, and length of stay supported the robustness of the key POD 1 side‐to‐side contrast.

**Conclusions:**

Orthopaedic trauma patients exhibit progressive early‐postoperative PPT reduction, with unilateral pain‐sensitivity alterations peaking on POD 1. Subsequent bilateral PPT decline may be compatible with broader postoperative changes in pressure pain sensitivity, but mechanistic sensitization cannot be inferred from PPT alone. Independent associations with perioperative PPT were identified for age, sex, and BMI in the adjusted model.

**Level of Evidence:**

Level III, prognostic study.

**Trial Registration:** Chinese Clinical Trial Registry: ChiCTR2500103937

## 1. Introduction

Pressure pain threshold (PPT) is an objective, quantitative sensory measure and intermediate biomarker reflecting pain sensitivity and nerve function and is widely applied in perioperative pain assessment among trauma patients [1–4]. For orthopaedic trauma patients, surgical intervention can induce local inflammatory responses and tissue injury, leading to dynamic fluctuations in PPT during the early postoperative period [5–7].

While previous studies have documented reduced PPT in injured extremities after orthopaedic surgery, several notable knowledge gaps persist [8, 9]. First, most prior research has neglected the comparison between affected and unaffected sides when analyzing PPT trajectories, failing to distinguish between surgery‐specific effects and systemic pain responses [10, 11]. Second, existing work predominantly depends on single postoperative time‐point assessments, and continuous bilateral PPT surveillance spanning the initial 72 postoperative hours remains limited [11–15]. Third, demographic and clinical variables are presumed to modulate PPT dynamics, yet comprehensive multivariable investigations are scarce, so their independent associations with perioperative PPT in patients with orthopaedic trauma have not been well characterized [8, 9, 16–18]. This knowledge gap hinders clinicians from optimizing perioperative care and improving patient recovery. Therefore, this longitudinal observational study aimed to characterize PPT alterations in trauma patients preoperatively and on postoperative days (PODs) 1, 2, and 3 and to identify independent associations with perioperative PPT.

## 2. Materials and Methods

### 2.1. Aim, Design, and Setting of the Study

This study aimed to characterize perioperative patterns of PPT alterations and identify independent associations with PPT to inform optimized pain control and early rehabilitation. This longitudinal observational study was conducted at the Department of Orthopaedics, Trauma Center, the Third Affiliated Hospital of Henan Medical University.

### 2.2. Participants

Eligible trauma patients were identified by querying the hospital’s electronic medical record system (EMRS) for patients admitted between June 16, 2025, and April 28, 2026, who underwent orthopaedic surgical intervention for acute traumatic injuries.

Inclusion criteria were defined as follows:

1. Aged 18–65 years old, with no sex restrictions.
2. Diagnosed with a single‐site acute traumatic injury (including fractures, dislocations, or soft tissue injuries requiring surgical management) of the extremities requiring surgical management.
3. Available longitudinal PPT data including preoperative baseline and postoperative follow‐up assessments.
4. No history of chronic pain disorders (e.g., rheumatoid arthritis and neuropathic pain) or prior surgical interventions at the same injury site.

Exclusion criteria were as follows:

1. Multiple traumatic injuries affecting two or more distinct anatomical sites.
2. Failure to undergo the predefined PPT monitoring schedule.
3. Systemic medical conditions with potential to alter pain‐processing function (e.g., diabetes mellitus with peripheral neuropathy and thyroid dysfunction).
4. Chronic regular use of analgesic or anti‐inflammatory agents prior to hospital admission.

The diagnosis of acute traumatic injuries was confirmed based on clinical manifestations, physical examination findings, and imaging results (X‐ray, computed tomography, or magnetic resonance imaging) by two attending orthopaedic surgeons independently. Discrepancies in diagnosis were resolved through a consensus meeting with a senior orthopaedic specialist.

### 2.3. Demographics and Study Population Characteristics

A total of 67 eligible patients were included in the final analysis. The mean age was 46.84 ± 16.07 years; there were 37 males (55.2%) and 30 females (44.8%). The mean body mass index (BMI) was 25.05 ± 4.24 kg/m^2^, with 5 underweight (BMI < 18.5 kg/m^2^, 7.46%), 28 normal (BMI 18.5–24.9 kg/m^2^, 41.79%), 28 overweight (BMI 25–29.9 kg/m^2^, 41.79%) and 6 obese (BMI ≥ 30 kg/m^2^, 9.00%). In terms of injury type, 38.81% (*n* = 26) of patients presented with upper extremity fractures, 58.21% (*n* = 39) with lower extremity fractures, and 2.99% (*n* = 2) with joint and soft tissue injuries requiring surgical debridement and repair. Regarding surgical approaches, 80.6% (*n* = 54) underwent open surgery, while 19.4% (*n* = 13) received minimally invasive surgical interventions. Anesthesia types included general anesthesia (*n* = 38, 56.7%), spinal/epidural anesthesia (*n* = 23, 34.3%), and regional nerve block anesthesia (*n* = 6, 9.0%).

### 2.4. Subject Recruitment Flow

As shown in Figure 1, a total of 124 trauma patients were initially retrieved from the hospital’s EMRS based on the study time frame and surgical criteria. Twenty‐eight were excluded for failing to meet the age requirements. The remaining 96 patients underwent rigorous eligibility evaluation per the predefined inclusion and exclusion criteria, with another 29 excluded: 17 had incomplete contralateral PPT monitoring data, 8 had a history of chronic pain, and 4 used long‐term analgesics before admission. Ultimately, 67 eligible patients were included in the final analysis.

**FIGURE 1:**
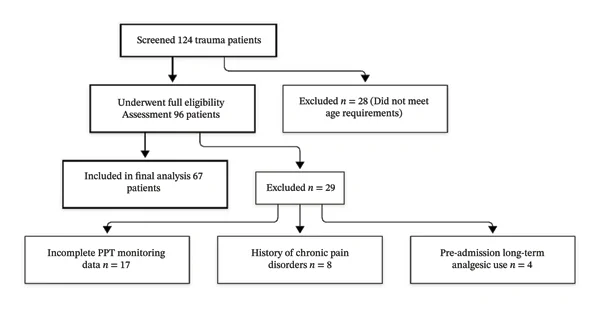
Participant flow diagram. A total of 124 trauma patients were initially screened. Of these, 28 were excluded for failing to meet the age criteria, 8 for a history of chronic pain, and 4 for long‐term analgesic use prior to admission. Another 17 patients were excluded due to incomplete pressure pain threshold data. Ultimately, 67 patients were enrolled in the final analysis.

### 2.5. PPT Measurement Protocol

PPT measurements were obtained using a calibrated electronic algometer (Model SH‐100; Yueqing Handpi Instruments Co., Ltd., Zhejiang, China) fitted with a probe with a contact area of 1 cm^2^. The device had a measurement resolution of 0.01 N and an accuracy of ±0.5%. The upper‐limb measurement site was defined as the midpoint of the line connecting the lateral epicondyle of the humerus and the cubital crease with the elbow flexed at 90°, and the lower‐limb site was located 10 cm proximal to the midpoint of the patellar base. All anatomical landmarks and contralateral mirror sites were marked on the skin with a sterile surgical marker during preoperative baseline assessment. The algometer was calibrated before each testing session and set to PEAK mode, with the LCD screen turned away from participants. Pressure was applied perpendicularly to the skin at a constant loading rate of 1 N/cm^2^/s. Pressure application was terminated immediately once participants reported the first perception of pain, and the peak force value was automatically recorded by the instrument. Three consecutive measurements were obtained at each marked site with a 1‐min interstimulus interval to prevent tissue accommodation and sequential pain sensitization. The mean value of the three measurements was adopted as the final PPT value [19–22]. All assessments were conducted by the same trained evaluator. Consistent supine positioning, probe orientation, loading rate, and standardized verbal instructions were maintained across all measurements. Bilateral PPT testing on affected and unaffected limbs was conducted at four time points: within 24 h preoperatively, when skin marking was completed; at 24 ± 2 h postoperatively on POD 1; on POD 2 using identical marked landmarks and testing parameters; and on POD 3 for the final assessment following the same protocol. All PPT measurements were carried out prior to scheduled analgesic administration whenever clinically feasible. The equipment and procedures for PPT measurement are detailed in Figure 2A.

**FIGURE 2:**
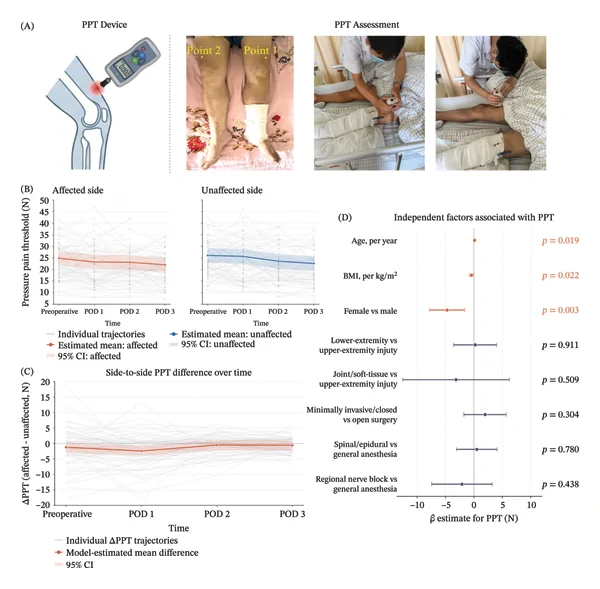
Measurement protocol, perioperative trajectories, side‐to‐side differences, and independent associations with PPT in orthopaedic trauma patients. (A) Experimental apparatus and PPT measurement procedures. PPT was measured bilaterally on the affected and unaffected sides using a calibrated electronic algometer at predefined perioperative time points. (B) Individual trajectories and model‐estimated marginal means of PPT on the affected and unaffected sides from the preoperative assessment to POD 3. Thin grey lines represent individual patient trajectories, colored lines represent model‐estimated marginal means, and shaded bands indicate 95% CI. (C) Longitudinal changes in side‐to‐side PPT difference, calculated as affected side minus unaffected side. Values below zero indicate lower PPT on the affected side relative to the unaffected side. (D) Forest plot of independent factors associated with PPT derived from LMM. Points represent *β* estimates, horizontal bars indicate 95% CI, and the vertical reference line indicates no association. Red markers denote statistically significant associations. PPT, pressure pain threshold; POD, postoperative day; CI, confidence interval; BMI, body mass index; LMM, linear mixed‐effects models.

### 2.6. Surgical and Treatment Procedures

All surgical interventions were performed by attending orthopaedic surgeons with ≥ 5 years of clinical experience in trauma surgery. Surgical approaches were categorized into two types based on clinical indications:

1. Open surgery: Applied for complex fractures with displacement or severe soft tissue injury, involving direct incision, exposure of the injury site, reduction, and fixation with plates, screws, or intramedullary nails.
2. Minimally invasive surgery: Selected for stable fractures or mild soft tissue injuries, using percutaneous fixation techniques with small incisions to minimize tissue trauma.

Anesthesia regimens were determined by the anesthesiologist according to the patient’s age, comorbidities, and surgical complexity:

1. General anesthesia: Anesthesia was induced with propofol (2–2.5 mg/kg) and maintained with sevoflurane (1.5–2.0 minimum alveolar concentration, MAC), combined with tracheal intubation for airway management.
2. Spinal anesthesia (subarachnoid block): Mainly indicated for surgeries involving the lower abdomen, pelvis, and lower extremities. The patient was placed in the lateral decubitus position with flexed knees and hips. Puncture was performed at the L3∼L4 or L2∼L3 intervertebral space. After successful puncture, 0.5% bupivacaine or 0.75% ropivacaine (1.5–2.5 mL) was injected. The anesthetic level was adjusted according to surgical requirements.
3. Regional nerve block anesthesia: Ultrasound‐guided peripheral nerve block was performed (e.g., femoral nerve block for lower extremity injuries, brachial plexus block for upper extremity injuries). 0.375% ropivacaine (20–30 mL) was administered, combined with intravenous sedation (midazolam 0.02–0.04 mg/kg) to improve patient comfort.

### 2.7. Postoperative Analgesic Management

Postoperative analgesic management followed routine institutional multimodal care and was individualized according to patient condition and clinician judgment. The regimen generally included nonsteroidal anti‐inflammatory drugs (NSAIDs), patient‐controlled intravenous analgesia, supplemental opioid or tramadol administration for breakthrough pain, and rescue analgesia when clinically required. Complete perioperative analgesic medication records were retrospectively reviewed. Opioid‐containing medications, including hydromorphone, butorphanol, and tramadol, were converted to cumulative morphine equivalent dose (MED) and used as an exploratory covariate in sensitivity analyses. Nonopioid analgesics were described but were not included in morphine‐equivalent conversion.

Physiotherapy and care:

1. Early mobilization: Range‐of‐motion exercises of joints within 24 h postoperatively; bed‐to‐chair transfers initiated on POD 1 for lower extremity injuries if fixation was stable.
2. Wound care: Body temperature was monitored daily, with routine inspection of surgical incisions for signs of infection.
3. Edema management: Cold compresses (20 min per session, 3 times daily) for the first 72 h postoperatively to reduce soft tissue swelling.

### 2.8. Outcome Measures

1. Monitoring Indicators: PPT measured on both the affected and unaffected sides at four time points (preoperative, POD 1–3), quantified in Newtons (N).
2. Primary Analysis: Longitudinal perioperative PPT changes across side and time using linear mixed‐effects models (LMM).
3. Key Planned Contrast: affected vs unaffected side on POD 1.
4. Potential Confounders: Age, sex, BMI, injury type, surgical approach, and anesthesia regimen.
5. Exploratory Clinical Variables: numerical rating scale (NRS) for pain intensity, cumulative MED, length of stay (LOS).

Data Sources and Bias Mitigation.

1. Data Sources: Demographic and clinical data were retrieved from the hospital’s EMRS; PPT measurements were recorded in a dedicated case report form (CRF).
2. Bias Reduction Strategies:
  - Measurement bias: All measurements were performed by a single trained assessor. The assessor was not involved in clinical care and was unaware of the study hypotheses but could not be blinded to the operated limb. Standardized measurement sites were marked preoperatively.
  - Selection bias: Strict predefined eligibility criteria applied uniformly; patient flow diagram documented exclusion reasons.
  - Information bias: Independent data extraction by two researchers; consensus resolution of discrepancies; standardized CRF for data collection.
  - Confounding bias: Controlled by eligibility criteria and adjusted in multivariable LMM where available.

### 2.9. Statistical Analysis

All statistical analyses were performed using Python software. Data processing and descriptive analyses were conducted using pandas and NumPy, LMM was fitted using statsmodels, and figures were generated using matplotlib. A two‐tailed *p* value < 0.05 was considered statistically significant. Continuous variables were presented as mean ± standard deviation or model‐estimated marginal means with 95% confidence intervals, as appropriate. Categorical variables were summarized as counts and percentages. For longitudinal analysis, PPT measurements were reorganized into long format. Each observation corresponded to one PPT measurement from a specific patient, side, and time point. LMM was used to assess longitudinal perioperative changes in PPT. Side, time, and their interaction were included as fixed effects. Age, BMI, sex, injury type, surgical approach, and anesthesia regimen were included to adjust for potential confounding effects. Patient ID was specified as a random intercept to account for repeated measurements and within‐subject correlation. Age and BMI were treated as continuous variables. Sex, injury type, surgical approach, and anesthesia regimen were treated as categorical variables, with male sex, upper‐extremity injury, open surgery, and general anesthesia used as reference categories. The model was fitted using restricted maximum likelihood estimation. Results were reported as *β* estimates, standard errors, 95% confidence intervals, and *p* values. Model‐estimated marginal means were calculated for each side and time point. Planned pairwise comparisons were conducted to evaluate interside differences at each time point, with the primary comparison defined as the affected versus unaffected side on POD 1. Within‐side postoperative changes were assessed by comparing each postoperative time point with the preoperative baseline. Side‐to‐side PPT difference was calculated as affected side minus unaffected side, with negative values indicating lower PPT on the affected side. Missing PPT observations were analyzed using all available repeated measurements in LMM, without imputation.

As an exploratory sensitivity analysis, cumulative MED, serial NRS pain scores, and LOS were additionally entered into LMM to examine whether the main PPT findings were robust to analgesic exposure, patient‐reported pain intensity, and hospitalization course. MED and LOS were treated as patient‐level exploratory variables, whereas NRS was treated as a time‐varying clinical pain variable. Detailed stepwise sensitivity models are provided in the Supporting Material.

A priori sample size calculation was performed using PASS 15.0 (NCSS, LLC, Kaysville, UT, USA). Using preliminary data, the required sample size was calculated assuming a mean paired difference of 1.8 units, a standard deviation of 4.7, a two‐sided *α* of 0.05, and a power of 0.80. Accounting for a 10% anticipated loss to follow‐up and potential outliers, the minimum calculated sample size was 59 subjects. Ultimately, 67 patients were enrolled to ensure sufficient statistical power for detecting significant interside and temporal changes in PPT.

## 3. Results

### 3.1. Model Construction and Available Observations

A total of 67 eligible orthopaedic trauma patients were included in the final analysis. Bilateral PPT measurements were scheduled at four perioperative time points, yielding a theoretical total of 536 repeated observations. After excluding unavailable PPT measurements, 526 valid observations were included in LMM.

The model was fitted using restricted maximum likelihood estimation and included patient ID as a random intercept to account for within‐subject correlation. Side, time, and their interaction were entered as fixed effects, with adjustment for age, BMI, sex, injury type, surgical approach, and anesthesia regimen. The model converged successfully. The mean number of observations per patient was 7.9, with a minimum of 5 and a maximum of 8 observations per patient.

### 3.2. Interside PPT Differences at Each Time Point

Adjusted interside comparisons (Table 1) showed that PPT on the affected side was consistently lower than that on the unaffected side across all perioperative time points. However, a statistically significant between‐side difference was observed only on POD 1.

**TABLE 1:** Adjusted comparison of pressure pain threshold between affected and unaffected sides at four time points.

Time point	Affected side, adjusted mean (*N*)	Unaffected side, adjusted mean (*N*)	Mean differences, affected–unaffected (*N*)	95% CI	*p* value
Preoperative	24.85	26.06	−1.21	−2.87–0.45	0.154
POD 1	23.22	25.64	−2.43	−4.08–−0.77	0.004
POD 2	23.07	23.54	−0.47	−2.17–1.22	0.583
POD 3	22.03	22.58	−0.55	−2.22–1.13	0.523

*Note:* POD, postoperative day.

Abbreviation: CI, confidence interval.

On POD 2 and POD 3, the between‐side differences were attenuated and no longer statistically significant. These findings suggest that the side‐to‐side PPT discrepancy was most pronounced on POD 1 and gradually diminished thereafter.

### 3.3. Perioperative Longitudinal Changes in PPT

The longitudinal trajectory analysis (Table 2) demonstrated a progressive decline in PPT on both the affected and unaffected sides during the first three PODs.

**TABLE 2:** Adjusted changes in PPT compared with preoperative baseline.

Side	Comparison	Mean differences (*N*)	95% CI	*p* value
Affected side	POD 1 vs Preoperative	−1.64	−3.29–0.02	0.053
	POD 2 vs Preoperative	−1.78	−3.46–−0.11	0.037
	POD 3 vs Preoperative	−2.82	−4.49–−1.15	< 0.001

Unaffected side	POD 1 vs Preoperative	−0.42	−2.08–1.25	0.623
	POD 2 vs Preoperative	−2.52	−4.20–−0.84	0.003
	POD 3 vs Preoperative	−3.48	−5.15–−1.81	< 0.001

*Note:* POD, postoperative day.

Abbreviation: CI, confidence interval.

On the affected side, relative to preoperative baseline, the PPT reduction at POD 1 approached but did not reach statistical significance, whereas significant PPT reductions were detected at POD 2 and POD 3.

On the unaffected side, no statistically significant PPT reduction was found at POD 1 versus baseline; significant decreases in PPT occurred at POD 2 and POD 3.

These results indicate that PPT reduction appeared earlier and more locally on the affected side, whereas the unaffected side showed a delayed but significant decline from POD 2 onward.

### 3.4. Side‐to‐Side PPT Difference Over Time

The side‐to‐side PPT difference was calculated as affected side minus unaffected side. A negative value therefore indicates lower PPT on the affected side relative to the unaffected side.

The adjusted side‐to‐side difference was −1.21 N preoperatively and became more pronounced on POD 1, reaching −2.43 N. Thereafter, the difference decreased to −0.47 N on POD 2 and ‐0.55 N on POD 3. This pattern suggests that the greatest lateralized pain sensitivity occurred on POD 1. The subsequent attenuation of the side‐to‐side difference was mainly attributable to a progressive decline in PPT on the unaffected side, rather than complete recovery of the affected side.

Although the overall time course showed numerically different patterns between the two sides, the side‐by‐time interaction terms were not statistically significant. Compared with the preoperative between‐side difference, the interaction estimates were 1.22 N on POD 1 (95% CI, −1.13 to 3.56; *p* = 0.309), −0.74 N on POD 2 (95% CI, −3.11 to 1.64; *p* = 0.544), and −0.66 N on POD 3 (95% CI, −3.03 to 1.70; *p* = 0.582). Therefore, while planned pairwise comparisons identified a significant between‐side difference on POD 1, the overall side‐by‐time interaction was not statistically significant.

### 3.5. Independent Associations With PPT

The multivariable linear mixed model identified age, sex, and BMI as variables with independent associations with PPT after adjustment for side, time, injury type, surgical approach, and anesthesia regimen (Table 3).

**TABLE 3:** Linear mixed‐effects model analysis of independent associations with PPT.

Factor	*β* estimate	SE	95% CI	*p* value
Age, per year	0.12	0.05	0.02–0.21	0.019
BMI, per kg/m^2^	−0.44	0.19	−0.82–−0.07	0.022
Female vs male	−4.72	1.57	−7.79–−1.64	0.003
Lower‐extremity vs upper‐extremity injury	0.22	1.93	−3.57–4.00	0.911
Joint/soft‐tissue vs upper‐extremity injury	−3.15	4.78	−12.53–6.22	0.509
Minimally invasive/closed vs open surgery	1.96	1.90	−1.77–5.69	0.304
Spinal/epidural vs general anesthesia	0.51	1.83	−3.07–4.09	0.780
Regional nerve block vs general anesthesia	−2.11	2.72	−7.44–3.22	0.438

Abbreviations: BMI, body mass index; CI, confidence interval; PPT, pressure pain threshold; SE, standard error.

Age was positively associated with PPT. Each 1‐year increase in age was associated with a 0.12 N increase in PPT, indicating relatively lower pressure pain sensitivity among older patients in this cohort.

Female sex was significantly associated with lower PPT. Compared with male patients, female patients had an adjusted PPT that was 4.72 N lower on average, suggesting higher pressure pain sensitivity in female patients.

BMI was also independently associated with PPT. Each 1 kg/m^2^ increase in BMI was associated with a 0.44 N decrease in PPT. This result indicates that higher BMI was related to increased pressure pain sensitivity.

In contrast, no statistically significant associations with PPT were observed for injury type, surgical approach, and anesthesia regimen.

### 3.6. Figure Description for Results Section

As shown in Figure 2B, individual PPT trajectories demonstrated substantial interindividual variability across the perioperative period. Nevertheless, model‐estimated marginal means showed a gradual postoperative decline in both affected and unaffected sides. Figure 2C further demonstrated that the side‐to‐side PPT difference was most evident on POD 1 and subsequently diminished over time. The forest plot in Figure 2D showed that age, sex, and BMI were independently associated with PPT, whereas no statistical associations with PPT were observed for injury type, surgical approach, and anesthesia regimen.

Exploratory sensitivity analyses were performed by additionally adjusting for cumulative MED, serial NRS pain scores, and LOS. The key affected‐versus‐unaffected side difference on POD 1 remained unchanged in the sensitivity model (mean difference, −2.43 N; 95% CI, −4.08 to −0.77; *p* = 0.004). In the fully adjusted model, MED, NRS, and LOS were not significantly associated with PPT. Adjustment for NRS attenuated some early within‐side postoperative time contrasts, particularly on the affected side, whereas the POD 3 reductions and the POD 1 side‐to‐side contrast remained consistent. NRS pain scores decreased from 4.91 ± 1.78 preoperatively to 2.27 ± 2.66 on POD 1, 1.81 ± 2.17 on POD 2, and 1.15 ± 1.51 on POD 3; however, NRS was not independently associated with PPT in the fully adjusted sensitivity model. Detailed outputs of the sensitivity analyses are provided in the Supporting Material.

## 4. Discussion

### 4.1. Principal Findings

In this longitudinal observational study, perioperative changes in PPT were characterized in orthopaedic trauma patients using repeated bilateral measurements and LMM. The principal findings were as follows. First, PPT showed a progressive decline during the first three PODs, indicating increased pressure pain sensitivity in the early postoperative period. Second, the affected side demonstrated significantly lower PPT than the unaffected side on POD 1, whereas the between‐side difference was attenuated on POD 2 and POD 3. Third, both the affected and unaffected sides showed significant reductions in PPT by POD 2 and POD 3. Finally, age, sex, and BMI were independently associated with PPT, whereas injury type, surgical approach, and anesthesia regimen were not significantly associated with PPT in the adjusted model.

These findings suggest a dynamic perioperative pattern of pressure pain sensitivity in trauma patients. In the immediate postoperative period, PPT reduction appeared to be more localized to the affected extremity. As the postoperative course progressed, the unaffected side also showed a significant decline in PPT, suggesting that early postoperative pain sensitivity may not remain confined to the injured or operated limb. The bilateral measurement design therefore provided clinically relevant information beyond a single‐site PPT assessment.

Exploratory sensitivity analyses supported the robustness of the POD1 side‐to‐side contrast.

### 4.2. Localized Pressure Pain Sensitivity on POD1

The most prominent interside difference was observed on POD 1, when PPT on the affected side was significantly lower than that on the unaffected side. Because lower PPT reflects greater pressure pain sensitivity [2, 23], this finding indicates pronounced local mechanical hyperalgesia in the affected limb during the immediate postoperative period. This early lateralized reduction in PPT may be related to local tissue injury, surgical incision, postoperative edema, alterations in peripheral nociceptor responsiveness, and mechanical stimulation around the injured and operated region [5, 13, 14].

Clinically, POD 1 may represent a critical window during which pain hypersensitivity is predominantly localized to the affected extremity. This finding supports careful local pain control, edema management, and cautious rehabilitation loading during the first 24 h after surgery. In particular, early rehabilitation should balance the benefits of mobilization with the risk of excessive stimulation in the affected limb when local pressure pain sensitivity is increased.

### 4.3. Bilateral PPT Decline and Broader Postoperative Changes in Pressure Pain Sensitivity

An important finding of this study was that the between‐side PPT difference diminished on POD 2 and POD 3. This attenuation should not be interpreted simply as recovery of the affected side, because PPT on the unaffected side also decreased significantly from POD 2 onward. In other words, the reduction in side‐to‐side discrepancy was partly attributable to a delayed decline in PPT on the contralateral, unaffected side.

The delayed reduction in PPT on the unaffected side may be compatible with a shift from localized postoperative hyperalgesia toward widespread alterations in pressure pain sensitivity. Surgical trauma and acute tissue injury may contribute to systemic inflammatory activation, perioperative stress responses, sleep disturbance, reduced mobility, and altered central nociceptive modulation [5, 9, 11, 14, 17, 23–25]. These factors may contribute to widespread changes in pressure pain sensitivity observed in the early postoperative period. Accordingly, bilateral PPT monitoring may facilitate discrimination between localized alterations in pain sensitivity affecting the operated limb and broader postoperative shifts in pressure pain sensitivity.

This interpretation is consistent with the observed trajectory: the affected side showed an early decreasing trend after surgery, whereas the unaffected side remained relatively stable on POD 1 but declined significantly on POD 2 and POD 3. These findings highlight that postoperative pain sensitivity is dynamic and may evolve over time. A single postoperative assessment may therefore be insufficient to capture the full pattern of perioperative pain sensitivity.

The exploratory sensitivity analyses provide additional context for interpreting PPT. NRS pain intensity decreased substantially during the early postoperative period, whereas PPT also showed a decreasing trend. This apparent divergence suggests that patient‐reported pain intensity and experimentally evoked mechanical pain sensitivity may capture different dimensions of perioperative pain. In the fully adjusted model, MED, NRS, and LOS were not independently associated with PPT. However, adjustment for NRS attenuated some early within‐side postoperative time contrasts, suggesting partial overlap between concurrent clinical pain intensity and short‐term PPT changes. Importantly, the POD 1 affected‐versus‐unaffected side difference remained stable after additional adjustment. These findings support interpreting PPT as a complementary sensory measure rather than a direct substitute for clinical pain scores.

### 4.4. Independent Associations With PPT

Age, sex, and BMI were independently associated with PPT after adjustment for side, time, injury type, surgical approach, and anesthesia regimen. Older age was associated with higher PPT values, suggesting relatively lower pressure pain sensitivity in older patients within this cohort. This association may be partly explained by age‐related changes in peripheral sensory function, mechanical perception, soft‐tissue characteristics, or pain reporting behavior [7, 15, 26]. However, the relationship between age and clinical pain is complex, and higher PPT should not be interpreted as uniformly lower postoperative pain burden in older patients.

Female sex was significantly associated with lower PPT compared with male sex, indicating greater pressure pain sensitivity among female patients. This finding is consistent with the broader pain literature showing sex‐related differences in experimental pain sensitivity [4, 13]. Potential mechanisms include hormonal regulation, endogenous pain inhibition, central nociceptive processing, psychosocial factors, and differences in body composition [4, 13, 26]. From a clinical perspective, female patients may require closer postoperative pain monitoring and more individualized analgesic and rehabilitation strategies.

Higher BMI was also independently associated with lower PPT. This finding indicates that patients with higher BMI showed greater pressure pain sensitivity in the present cohort. Possible explanations include increased mechanical loading, obesity‐related low‐grade inflammation, altered soft‐tissue pressure distribution, and reduced postoperative mobility [9, 11, 27–29]. Nevertheless, inflammatory biomarkers and body composition parameters were not measured in this study; therefore, the mechanism underlying the association between BMI and PPT requires further investigation.

Taken together, these results suggest that demographic and body‐composition characteristics should be considered when interpreting PPT measurements. PPT values should not be viewed as purely reflecting local injury severity; they may also be influenced by individual biological and sensory characteristics. Incorporating these factors into perioperative assessment may improve the identification of patients with heightened pain sensitivity.

### 4.5. Injury Type, Surgical Approach, and Anesthesia Regimen

In contrast, injury type, surgical approach, and anesthesia regimen were not significantly associated with PPT in the adjusted model. This finding suggests that, within the present cohort, individual patient characteristics may have stronger associations with PPT than broad surgical or anesthetic categories. However, these null findings should be interpreted cautiously. Some subgroups, such as joint or soft‐tissue injuries and regional nerve block anesthesia, included relatively few patients, which may have limited statistical power.

In addition, surgical approach and anesthesia regimen were determined according to clinical indications rather than random allocation. Therefore, residual confounding cannot be excluded. The absence of statistically significant associations should not be interpreted as evidence that surgical trauma severity or anesthetic strategy has no influence on postoperative pain sensitivity. Future studies with larger and more balanced subgroups are needed to clarify these relationships.

### 4.6. Clinical Implications

The present findings have several clinical implications. First, the significant side‐to‐side difference on POD 1 indicates that the first POD may be a key period for targeted local analgesia, edema control, and careful rehabilitation load management of the affected extremity. Second, the bilateral decline in PPT from POD 2 onward suggests that postoperative pain management should not focus solely on the injured limb but should also acknowledge bilateral changes in pressure pain sensitivity during the early postoperative course. Third, the independent associations of age, sex, and BMI with PPT highlight the importance of individualized interpretation of quantitative sensory testing results. Patients with lower PPT may require closer monitoring, more proactive analgesic adjustment, and individualized rehabilitation progression. Repeated PPT monitoring may provide an objective supplement to patient‐reported pain intensity and may help clinicians better understand perioperative pain sensitivity in trauma patients.

The sensitivity analysis incorporating serial NRS, cumulative MED, and LOS provides insights for perioperative PPT evaluation. It suggests that the interpretation of PPT outcomes should be contextualized with clinical pain status and perioperative analgesic exposure. Early local reductions in PPT should therefore be interpreted cautiously and cannot be simply attributed to persistent surgical trauma‐induced hyperalgesia. By comparison, interside PPT comparisons across all time points are less susceptible to postoperative pain interference, representing a relatively stable indicator for evaluating limb sensory differences after orthopaedic trauma surgery. Notably, the current study does not provide evidence to support that PPT can predict hospital stay, analgesic requirements, or rehabilitation outcomes.

### 4.7. Strengths and Limitations

This study has several strengths. Bilateral PPT assessment allowed each patient to serve partially as their own internal control, improving the interpretation of side‐to‐side differences. Repeated measurements from the preoperative period to POD 3 enabled characterization of early postoperative PPT trajectories. In addition, the use of LMM accounted for within‐subject correlations and allowed all available repeated observations to be included in the analysis.

Several limitations should also be acknowledged. First, this was a single‐center observational study with a relatively small sample size, which may limit the generalizability of the findings. Second, the study cohort exhibited substantial heterogeneity with respect to injury type, surgical approach, and anesthesia regimen. In addition, several clinical subgroups were small—notably patients with joint or soft‐tissue injuries and individuals receiving regional nerve block anesthesia—thereby limiting the statistical power to identify potential subgroup differences. Although LMM was used to adjust for measured confounders, residual confounding stemming from cohort heterogeneity could not be fully eliminated. Third, although LMM allowed inclusion of all available repeated measurements, a small number of PPT observations were missing, and potential missing‐data bias cannot be fully excluded. Fourth, the follow‐up period was limited to the first three PODs; moreover, outcomes such as quality of recovery, functional performance, and postoperative complications were not collected, and long‐term follow‐up was absent. Accordingly, definitive clinical predictive value of PPT could not be established in the present cohort. Fifth, although the assessor performing PPT measurements was uninvolved in clinical care and unaware of the study hypotheses, complete blinding to the operated limb could not be achieved. This unavoidable limitation may introduce potential measurement bias. Sixth, analgesic management was not a protocol‐driven study intervention. While standardized multimodal analgesia was applied, analgesic regimens were individually adjusted according to routine clinical judgment, which may introduce unmeasured bias. Cumulative MED was derived retrospectively and could not capture the full spectrum of analgesic exposure; specifically, nonopioid rescue medications including NSAIDs were not incorporated into MED conversion. Seventh, NRS pain scores were extracted from routine clinical records and compiled retrospectively, rather than collected under a strict prospective outcome‐driven study design. Finally, psychological factors, inflammatory biomarkers, detailed rehabilitation intensity, and sleep quality were not included, although these variables may influence pain sensitivity [9, 14, 29–31].

### 4.8. Future Directions

Future studies should include larger multicenter cohorts with longer follow‐up to determine whether early postoperative PPT trajectories predict persistent postoperative pain, analgesic requirements, and functional recovery. Integrating PPT with patient‐reported pain scores, analgesic consumption, inflammatory biomarkers, psychological assessments, and rehabilitation outcomes may further clarify the mechanisms driving postoperative alterations in pain sensitivity and support the development of personalized perioperative pain management strategies.

### 4.9. Conclusion of Discussion

Overall, this study demonstrates that PPT decreases dynamically during the early postoperative period in orthopaedic trauma patients. The affected side shows the most prominent pressure pain sensitivity on POD 1, whereas subsequent bilateral PPT decline may be compatible with broader postoperative changes in pressure pain sensitivity. Age, sex, and BMI show independent associations with PPT levels and should be considered when interpreting perioperative PPT measurements. PPT may provide an objective supplement to clinical pain assessment and may help inform individualized management and early rehabilitation planning in orthopaedic trauma patients.

**Nomenclature**
PPT: Pressure pain threshold
BMI: Body mass index
PASS: Power analysis and sample size
EMRS: Electronic medical record system
CRF: Case report form
POD: Postoperative day
MAC: Minimum alveolar concentration
LMM: Linear mixed‐effects models
NSAIDs: Nonsteroidal anti‐inflammatory drugs
MED: Morphine equivalent dose
NRS: Numerical rating scale
LOS: Length of stay

## Author Contributions

Zhijie Yan and Hao Li designed the study, collected clinical data, drafted, and revised the manuscript. Ziyu Wang, Wuzhen Su, Yibin Gao, Ruiyun Li, Chunwang Zhang, and Zhicheng Gao participated in patient enrollment, PPT measurement, and data sorting. Hewei Wang and Jie Liu conceived the study, supervised the research work, and revised the manuscript critically.

## Funding

This work was supported by the Young Scientists Fund of the Natural Science Foundation of Henan Province (Grant No. 252300420635) and the 2025 Henan Provincial Medical Science and Technology Talent Overseas Training Program (Grant No. HNMOT2025082).

## Disclosure

All authors have read and approved the final manuscript. All content, viewpoints, data interpretations, and conclusions were fully reviewed, revised, and verified by the authors. The authors take full responsibility for the integrity and accuracy of the entire manuscript.

## Ethics Statement

This study was conducted in strict accordance with the ethical principles outlined in the 1964 Declaration of Helsinki and its subsequent amendments. Ethical approval was obtained from the Ethics Committee of the Third Affiliated Hospital of Henan Medical University (approval no. K2025‐026–01).

## Consent

The authors have nothing to report.

## Conflicts of Interest

The authors declare no conflicts of interest.

## Supporting Information

Additional supporting information can be found online in the Supporting Information section.

## Supporting information


**Supporting Information** Supporting materials.docx: Contains supporting methods for sensitivity linear mixed‑effects models, Supporting Table 1 (adjusted comparison of PPT between affected and unaffected sides at four time points in the sensitivity model), Supporting Table 2 (stratified sensitivity analyses for within‑side PPT changes), Supporting Table 3 (independent associations from the full sensitivity model), Supporting Table 4 (descriptive statistics of NRS pain scores at each perioperative time point), Supporting Table 5 (adjusted changes in NRS compared with preoperative baseline), and Supporting Table 6 (equianalgesic conversion ratios for intravenous opioids to morphine‑equivalent dose).

## Data Availability

The datasets used and/or analyzed during the current study are available from the corresponding authors on reasonable request.
