# Supplementary material for: Perioperative Dynamic Changes and Independent Associations of Pressure Pain Threshold in Trauma Patients: A Prospective Longitudinal Study
Source: Pain Res Manag. 2026 Sep 29;2026:8214001. doi: 10.1155/prm/8214001 (PMC13621237; doi:10.1155/prm/8214001)
Supplement: Supplementary file 1 [file PRM-2026-8214001-s001.docx]

**Supplementary materials**

The following supplementary analyses were performed to examine the robustness of the main pressure pain threshold (PPT) findings after additional adjustment for postoperative clinical variables. These analyses were exploratory and were not intended to replace the primary linear mixed-effects models (LMM). Cumulative morphine equivalent dose (MED) and length of stay (LOS) were treated as patient-level exploratory variables, whereas numerical rating scale (NRS) was treated as a time-varying pain intensity measure. Because analgesic management was not randomized or experimentally controlled, these analyses should not be interpreted causally.

Supplementary Methods: To assess the robustness of primary model findings and reduce postoperative confounding bias, a full sensitivity LMM was first constructed. This sensitivity model retained all fixed-effect terms, random intercept structure, and reference category settings from the primary model, with three clinically relevant postoperative covariates added simultaneously: cumulative MED, serial NRS pain scores, and LOS. The consistency of effect direction, magnitude, and statistical significance between the primary model and full sensitivity model was compared to preliminarily judge the stability of core results. Where notable shifts in statistical significance were observed after simultaneous adjustment for all three postoperative covariates, hierarchical sequential sensitivity analyses were further performed to identify the key confounding variable driving such changes. Four nested stepwise models were established: Model 0 (primary model with only baseline confounders, no postoperative covariates), Model 01 (primary model plus MED alone), Model 02 (primary model plus MED and NRS, and Model 03 (full sensitivity model incorporating MED, NRS and LOS together). Shifts in PPT effect sizes and P values were tracked as covariates were added incrementally across hierarchical models.

**Supplementary Table 1. Adjusted comparison of PPT between affected and unaffected sides at four time points in the sensitivity model**

| **Time point** | **Mean differences, affected - unaffected (N)** | **95% CI** | **P value** |
| --- | --- | --- | --- |
| Preoperative | -1.21 | -2.88 to 0.45 | 0.153 |
| POD 1 | -2.43 | -4.08 to -0.77 | 0.004 |
| POD 2 | -0.48 | -2.17 to 1.22 | 0.583 |
| POD 3 | -0.54 | -2.22 to 1.13 | 0.525 |

*PPT, pressure pain threshold; POD, postoperative day; CI, confidence interval.*

**Supplementary Table 2. Stratified sensitivity analyses for within-side PPT changes**

| **Side** | **Comparison** | **Model 0 (Primary model)**  **β (95% CI), P** | **Model 01 (+MED)**  **β (95% CI), P** | **Model 02 (+MED+NRS)**  **β (95% CI), P** | **Model 03 (+MED+NRS+LOS)**  **β (95% CI), P** |
| --- | --- | --- | --- | --- | --- |
| Affected | POD1 vs Pre | −1.635 (−3.291, 0.021), P=0.053 | −1.635 (−3.291, 0.021), P=0.053 | −1.317 (−3.140, 0.505), P=0.157 | −1.319 (−3.142, 0.503), P=0.156 |
| Affected | POD2 vs Pre | −1.785 (−3.463, −0.106), P=0.037 | −1.784 (−3.462, −0.106), P=0.037 | −1.409 (−3.313, 0.495), P=0.147 | −1.410 (−3.313, 0.494), P=0.147 |
| Affected | POD3 vs Pre | −2.816 (−4.487, −1.146), P<0.001 | −2.815 (−4.486, −1.145), P<0.001 | −2.360 (−4.355, −0.365), P=0.020 | −2.362 (−4.357, −0.367), P=0.020 |
| Unaffected | POD1 vs Pre | −0.415 (−2.078, 1.248), P=0.625 | −0.415 (−2.078, 1.248), P=0.625 | −0.101 (−1.926, 1.725), P=0.914 | −0.104 (−1.930, 1.722), P=0.911 |
| Unaffected | POD2 vs Pre | −2.520 (−4.203, −0.837), P=0.003 | −2.519 (−4.202, −0.836), P=0.003 | −2.145 (−4.052, −0.238), P=0.027 | −2.147 (−4.054, −0.240), P=0.027 |
| Unaffected | POD3 vs Pre | −3.477 (−5.148, −1.807), P<0.001 | −3.477 (−5.148, −1.807), P<0.001 | −3.027 (−5.016, −1.038), P=0.003 | −3.030 (−5.020, −1.041), P=0.003 |

*PPT, pressure pain threshold; POD, postoperative day; CI, confidence interval; MED, morphine equivalent dose; NRS, numerical rating scale; LOS, length of stay.*

**Supplementary Table 3. Independent associations from the full sensitivity model**

| **Factor** | **β estimate** | **SE** | **95%CI** | **P-value** |
| --- | --- | --- | --- | --- |
| Age, per year | 0.14 | 0.05 | 0.04 to 0.23 | 0.004 |
| BMI, per kg/m² | -0.42 | 0.21 | -0.84 to -0.01 | 0.044 |
| Female vs male | -4.73 | 1.58 | -7.83 to -1.63 | 0.003 |
| MED | -0.00 | 0.02 | -0.03 to 0.03 | 0.954 |
| NRS | 0.12 | 0.15 | -0.17 to 0.41 | 0.418 |
| LOS | -0.04 | 0.11 | -0.25 to 0.17 | 0.700 |

*BMI, body mass index; MED, morphine equivalents dose; NRS, numerical rating scale; LOS, length of stay; SE, standard error, CI, confidence interval.*

**Supplementary Table 4. Descriptive statistics of NRS pain scores at each perioperative time point**

| **Time point** | **Mean±SD** | **Median** | **Rang** |
| --- | --- | --- | --- |
| Preoperative | 4.91±1.78 | 5 | 0 to 9 |
| POD1 | 2.27±2.66 | 1 | 0 to 10 |
| POD2 | 1.81±2.17 | 0 | 0 to 8 |
| POD3 | 1.15±1.51 | 0 | 0 to 5 |

*NRS, numerical rating scale; POD, postoperative day; SD, standard deviation.*

**Supplementary Table 5. Adjusted changes in NRS compared with preoperative baseline**

| **Side** | **Comparison** | **Mean differences (N)** | **P-value** |
| --- | --- | --- | --- |
| Affected side | POD1 vs Preoperative | -2.62 | <0.001 |
|  | POD2 vs Preoperative | -3.07 | <0.001 |
|  | POD3 vs Preoperative | -3.75 | <0.001 |

*NRS, numerical rating scale; POD, postoperative day.*

**Supplementary** **Table 6. Equianalgesic conversion ratios for intravenous opioids to MED**

| **Intravenous analgesic agent** | **Unit of drug** | **Equivalent intravenous morphine dose** |
| --- | --- | --- |
| Hydromorphone hydrochloride | 1 mg | 10 mg |
| Butorphanol tartrate | 1 mg | 5 mg |
| Tramadol | 1 mg | 0.1 mg |

*Equianalgesic ratios were adopted from widely recognized clinical reference standards to calculate cumulative perioperative MED. MED was incorporated as a continuous covariate in linear mixed-effects models sensitivity analyses. Non‑opioid analgesic agents, including non‑steroidal anti‑inflammatory drugs, were not incorporated into MED conversion. MED, morphine equivalent dose.*
